# Avirulent phenotype promotes *Bordetella pertussis* adaptation to the intramacrophage environment

**DOI:** 10.1080/22221751.2022.2146536

**Published:** 2023-01-19

**Authors:** Mariam R. Farman, Denisa Petráčková, Dilip Kumar, Jakub Držmíšek, Argha Saha, Ivana Čurnová, Jan Čapek, Václava Hejnarová, Fabian Amman, Ivo Hofacker, Branislav Večerek

**Affiliations:** a Institute for Theoretical Chemistry, University of Vienna, Vienna, Austria; b Czech Academy of Sciences, Laboratory of Post-transcriptional Control of Gene Expression, Institute of Microbiology, Prague, Czech Republic

**Keywords:** *Bordetella pertussis*, BvgAS, intramacrophage environment, adaptation to stress, cysteine toxicity, avirulent phase

## Abstract

*Bordetella pertussis*, the causative agent of whooping cough, is an extracellular, strictly human pathogen. However, it has been shown that *B. pertussis* cells can escape phagocytic killing and survive in macrophages upon internalization. Our time-resolved RNA-seq data suggest that *B. pertussis* efficiently adapts to the intramacrophage environment and responds to host bactericidal activities. We show that this adaptive response is multifaceted and, surprisingly, related to the BvgAS two-component system, a master regulator of virulence. Our results show that the expression of this regulatory circuit is downregulated upon internalization. Moreover, we demonstrate that the switch to the avirulent Bvg^−^ phase augments a very complex process based on the adjustment of central and energy metabolism, cell wall reinforcement, maintenance of appropriate redox and metal homeostasis, and repair of damaged macromolecules. Nevertheless, not all observed effects could be simply attributed to the transition to Bvg^−^ phase, suggesting that additional regulators are involved in the adaptation to the intramacrophage environment. Interestingly, a large number of genes required for the metabolism of sulphur were strongly modulated within macrophages. In particular, the mutant lacking two genes encoding cysteine dioxygenases displayed strongly attenuated cytotoxicity toward THP-1 cells. Collectively, our results suggest that intracellular *B. pertussis* cells have adopted the Bvg^−^ mode to acclimate to the intramacrophage environment and respond to antimicrobial activities elicited by THP-1 cells. Therefore, we hypothesize that the avirulent phase represents an authentic phenotype of internalized *B. pertussis* cells.

## Introduction

*Bordetella pertussis* is a strictly human reemergent pathogen of the respiratory tract and the etiologic agent of whooping cough [1]. This highly contagious disease is particularly severe in infants and remains a major cause of infant mortality worldwide, especially in developing countries [2].

*B. pertussis* has been traditionally described and viewed as an extracellular pathogen that colonizes the epithelium of the upper respiratory tract (recently reviewed in [3]). While ciliated epithelial cells secrete mucus that mechanically eliminates the bacteria, both resident and attracted phagocytic cells interact with the pathogen to clear the infection [4]. Successful infection, therefore, depends on the activities of a variety of virulence factors produced by *B. pertussis* cells [3]. In particular, adhesins such as filamentous haemagglutinin and fimbriae contribute to efficient adhesion and colonization of the epithelium, while toxins such as pertussis toxin and adenylate cyclase toxin disrupt the epithelium and modulate the activity of the immune system, thereby facilitating the survival of bacteria in the respiratory tract [3,5,6]. The expression of most virulence factors is controlled by the two-component system BvgAS, which consists of the sensor kinase BvgS and the response regulator BvgA, which upon phosphorylation with BvgS promotes expression of virulence-activated genes [7]. Basically, three different phenotypes can be distinguished based on the BvgA phosphorylation status: Bvg^+^ (virulent), Bvg^−^ (avirulent) and Bvg^i^ (intermediate). However, the exact role of the avirulent phase in the infection cycle of *B. pertussis* remains enigmatic [8-10].

In the presence of specific antibodies, opsonized *B. pertussis* cells are internalized and killed by phagocytic cells [11,12]. However, some early reports suggested that in the absence of opsonizing antibodies, this pathogen stimulates its own attachment to immune cells, resulting in inefficient killing by phagocytes and increased intracellular survival [12-16]. Importantly, *B. pertussis* cells have also been found *in vivo* in human, rabbit and mouse alveolar macrophages [17-19]. In the last decade, Rodriguez and her team have presented data showing that *B. pertussis* can survive in primary macrophages [20] as well as in monocyte-derived THP-1 macrophages [21,22]. They have shown that most *B. pertussis* cells are killed within acidic compartments, but some residual cells escape this killing and proliferate in a nonacidic, early endosomal compartment. These data led to speculation that *B. pertussis* may utilize macrophages as an intracellular niche and that the intramacrophage phase of infection may play a significant role in the *B. pertussis* infection cycle [14,18,19]. In support of this hypothesis, gene expression profiling and proteomic analysis of infected monocyte-derived THP-1 macrophages revealed that intracellular *B. pertussis* cells modulate the expression of several genes involved in the host immune response and reshape the production of dozens of proteins implicated in virulence [21-23]. Furthermore, Harvill and colleagues have demonstrated that the closely related pathogens *B. bronchiseptica* and *B. parapertussis* survive intracellularly in amoebae [24] and primary macrophages [25], respectively. These observations suggest that the ability for intracellular survival has an ancestral origin and is widespread among pathogenic *Bordetella* species [26,27].

To date, transcriptional profiling of *B. pertussis* interactions with host cells has been studied primarily in mouse models and has focused on either the host [28,29] or the pathogen [30,31]. Moreover, these studies did not address the importance of the intracellular phase in the infection cycle of *B. pertussis*. Therefore, we recently engaged the dual RNA-seq method to analyze parallel transcriptomic profiles of human macrophages infected with *B. pertussis* to elucidate the nature of the interactions between the pathogen and the immune cell [23]. To avoid the donor-to-donor variation observed in experiments with primary macrophages, we used THP-1 monocyte-derived cells, a popular cell model for immune modulations studies [32] that has been shown to be a good surrogate for primary cells in experiments with *B. pertussis* [22]. In the pilot experiment, which focused primarily on host cells, we demonstrated that in response to *B. pertussis* infection, the global gene expression profiles of THP-1 macrophages were extensively rewired during the course of infection [23]. Importantly, we have also shown that significant numbers of bacterial reads can be obtained from infected macrophage cells, provided we focus on the early stages of infection and substantially increase the sequencing depth.

In this manuscript, we focused on determining temporal changes in gene expression profiles of intracellular *B. pertussis* cells using time-resolved dual RNA-seq analysis. Our results indicate that intracellular *B. pertussis* cells switch from a virulent to an avirulent phenotype, which appears to be beneficial for intramacrophage survival and persistence, and support the emerging hypothesis that the avirulent phase represents an authentic phenotype of intracellular *B. pertussis* cells.

## Materials and methods

### Cultivation of *B. pertussis* cells

The *Bordetella pertussis* reference strain Tohama I and its derivatives (Table S3) were grown on Bordet-Gengou (BG) agar plates (Difco) supplemented with 15% sheep blood for 3 days at 37°C. For liquid cultures, cultures were inoculated from agar plates and grown in Stainer-Scholte (SS) medium [33] supplemented with 0.1% cyclodextrin (Sigma) and 0.5% casamino acids (Difco) at 37°C. For infection assays, the *B. pertussis* cells were grown overnight in SS medium to the mid-exponential phase of growth (OD_600_ ≈ 1) and diluted to the appropriate cell density in RPMI medium.

### Construction of *B. pertussis* mutants

The deletions were introduced into the chromosome of the *B. pertussis* Tohama I strain as described previously [34]. To construct the Δ*BP2871* deletion mutant, two DNA fragments of approximately 750 bp flanking the corresponding gene were amplified from the upstream region (which ends with the ATG codon of the Δ*BP2871* gene and the *Spe*I site) and the downstream region (which begins with the *Spe*I site and the TGA stop codon of the Δ*BP2871* gene). The resulting PCR products were ligated via a *Spe*I site, and the ligation mixture was used as a template to generate a PCR product of approximately 1.5 kb containing the intended deletion. In the resulting product, the start and stop codons of the corresponding gene separated by the *Spe*I restriction site formed a markerless in-frame deletion. The Δ*BP3011* deletion was constructed in the same manner, but the *Nhe*I site was used instead of *Spe*I to ligate both DNA fragments. In all cases, the final PCR products were ligated into the allelic exchange plasmid pSS4245 and cloned into the *E. coli* strain XL1-Blue. Finally, the resulting recombinant plasmid was transformed into the *E. coli* SM10 strain (donor strain) and transferred to *B. pertussis* Tohama I (recipient strain) by conjugation, as described elsewhere [35]. After two recombination events, the strain with the desired deletion was obtained. To create Δ*BP2871*Δ*BP3011* double mutant, the Δ*BP2871* mutant was conjugated with pSS4245 derivative carrying the Δ*BP3011* allele. All mutations were confirmed by sequencing the amplified PCR product covering the regions adjacent to the deletion. The plasmids and primers used in this study are listed in Table S2 and Table 3, respectively.

### Growth and differentiation of THP-1 cells

Monocytic THP-1 cells (ATCC; TIB-202) were grown in suspension in tissue culture-treated dishes (100 mm; Corning) in Roswell Park Memorial Institute (RPMI) 1640 medium (Sigma, R8758) supplemented with 10% heat-inactivated fetal bovine serum (Sigma) at 37°C in a humidified incubator (5% CO_2_). For differentiation into macrophages, THP-1 monocytes were seeded in 6-well plates (4 × 10^6^ cells per well) and stimulated by the addition of 100 nM phorbol 12-myristate 13-acetate (Sigma) for 72 h, followed by a 24-h resting period in plain RPMI medium.

### Infection of THP-1-derived macrophages

Differentiated THP-1 macrophages were infected with *B. pertussis* cells diluted in RPMI medium at a multiplicity of infection of 10 bacteria per macrophage (4 × 10^7^ per well). The plates were then centrifuged at 600 g for 3 min to facilitate the interaction of bacteria with macrophages. After 1 h of incubation (37°C; 5% CO_2_), extracellular bacteria were removed by washing with RPMI medium, and the remaining bacteria were killed by incubation in RPMI medium containing 100 μg/ml of polymyxin B sulphate (Sigma) for 1 h (37°C; 5% CO_2_). No viable bacteria were detected by direct plating of cell culture supernatants on BG agar (data not shown). From this time point (T2), corresponding to 2 h pi (1 h of co-incubation of macrophages with *B. pertussis* cells and 1 h of polymyxin B treatment), the concentration of antibiotic was maintained at 25 μg/ml which was sufficient to prevent repopulation of the RPMI medium with released intracellular bacteria. To obtain samples of infected macrophages 1 h pi (T1), both co-incubation of *B*. *pertussis* with THP-1 cells and treatment with polymyxin B were performed for 30 min each. Again, no viable bacteria were detected by direct plating of cell culture supernatants on BG agar. At the indicated time points (1, 2, 4, 8 and 12 h pi; corresponding to time points T1–T5), infected cells were washed intensely with prewarmed phosphate-buffered saline (PBS) and directly lysed. In parallel, samples of uninfected macrophages treated in the same manner and samples of intact bacteria incubated in RPMI at 37°C served as controls. In addition, at each time point infected macrophages from a separate well were lysed with sterile water containing 0.01% Triton X-100, and the lysates were plated onto BG agars to determine the numbers of viable intracellular bacteria. At each time point, three biological replicates of infected and uninfected macrophages and unexposed bacteria were harvested for RNA isolation.

### RNA isolation

Samples of biological triplicates of infected and uninfected macrophages were lysed at each time point with 0.5 ml of TRI reagent (Sigma), and the lysates were stored at −80°C. Total RNA was isolated from the lysates according to the manufactureŕs protocol. Cultures of *B. pertussis* cells grown in biological triplicates in RPMI medium (controls) were mixed 4:1 with stop solution (95% ethanol, 5% phenol), pelleted, and stored at −80°C. Bacterial pellets were suspended in TE buffer (10 mM Tris, 1 mM EDTA; pH 8.0) containing 1 mg/ml lysozyme (Sigma) and total RNA was isolated from lysed cells using TRI reagent (Sigma) according to the manufacturer’s protocol. DNA was removed by treatment of all samples with the TURBO DNA-free kit (Thermo Fisher Scientific). Quality and quantity of RNA were determined by agarose gel electrophoresis and using the Nanodrop 2000 instrument (Thermo Fisher Scientific). In addition, RNA quality was assessed at the sequencing facility (Vienna Biocenter Core Facility, NGS unit) on an Agilent 2100 Bioanalyzer. All samples displayed RNA integrity numbers greater than 9.

### Library preparation, sequencing, read mapping and DE analysis

Host and pathogen ribosomal RNAs were simultaneously depleted using the Ribo-Zero Gold rRNA Removal Kit (Illumina). Libraries were prepared using the NEBNext® Ultra™ II DNA Library Prep Kit for Illumina and sequenced on an Illumina HiSeq 2500 platform using HiSeqV4 chemistry with single-end 100-base-pair reads. FastQC (https://www.bioinformatics.babraham.ac.uk/projects/fastqc; version 0.11.9) was used to perform quality control checks on raw Illumina reads in FASTQ format. A single reference transcriptome generated by combining the human transcriptome (Ensembl GRCh38) and *B. pertussis* Tohama I transcriptome was used to map and quantify reads with Salmon quantifier (version 1.1.0) [36]. The *B. pertussis* reference transcriptome sequence file was generated using the gffread tool (https://github.com/gpertea/gffread), the Tohama I reference sequence (GCF 000195715.1) from the NCBI assembly, and the corresponding Tohama I gene annotation in GFF file format (GCF_000195715.1_ASM19571v1_genomic.gff). After quantification performed with Salmon and the reference transcriptome file, the human and bacterial read counts per gene were separated for subsequent differential expression analysis. Unwanted variations caused by batch effects or library preparation were removed from the samples prior to DE analysis using the RUVs correction method of RUVseq (version 1.24.0) [37] in R (version 4.0.5). DE analysis was performed with the EdgeR package (version 3.32.1) [38] using the gene read counts as inputs. To normalize for sequencing depth, we calculated library size normalization factors for each library using the weighted Trimmed Mean of M-values method implemented in the EdgeR by means of calcNormFactors function.

### Bioinformatics analyzes

To perform principal component analysis (PCA) for THP-1 macrophages and *B. pertussis* samples, read counts per gene were first normalized by regularized log transformation implemented in the rlog function of DESeq2 [39] for each time point. The plots were generated using the ggfortify (https://github.com/sinhrks/ggfortify; version 0.4.14) and ggplot2 (version 3.3.5) [40] packages in R. GO term enrichment analysis for *B. pertussis* genes was deduced using blast2go [41]. The log fold changes obtained from DE analysis were used for each gene and sample for the corresponding term. Each GO term associated with more than one gene was tested for enrichment compared to the whole transcriptome using Fisher’s exact test. Subsequently, the determined *p*-values were corrected for multiple testing by the method of Benjamini and Hochberg [42]. The 20 most enriched “Upregulated” and “Downregulated” gene sets for each time point were selected for visualization. The package ggplot2 was used in R to generate heatmaps for the selected terms and corresponding genes for the five infection time points.

### Validation of the RNA-seq data by quantitative PCR

To validate the gene expression profiles obtained from RNA-seq analysis, the expression of selected *B. pertussis* genes was examined by RT-qPCR method using the same RNA samples previously used for RNA-seq analysis. Isolated RNA (600 ng) was reverse transcribed into cDNA using random hexamers and M-MLV reverse transcriptase (Promega) at 37°C in a 25-μl reaction according to the manufacturer’s instructions. RT-qPCR reactions were performed in at least three technical replicates per sample on the Bio-Rad CFX96 instrument using SYBR® Green JumpStart™ Taq ReadyMix™ (Sigma), 4 pmoles of each primer, and 2 µl of fiftyfold diluted cDNA in a 20-μl reaction. Each reaction consisted of an initial step at 95°C for 2 min, 40 cycles of 95°C for 15 s, 65°C for 30 s, and 72°C for 30 s, followed by a melting curve recording. The sequences of primers used for gene expression analysis of THP-1 and *B. pertussis* have been published previously [23,34,43] or are listed in Table S3. The *rbfA* gene was used as a reference gene to determine relative gene expression in intracellular and unexposed bacteria. Relative gene expression was determined using the delta-delta C_t_ method [44].

### THP-1 cells viability assay

The viability of infected THP-1 macrophages was determined spectrophotometrically as the capacity of mitochondrial dehydrogenase to induce cleavage of the tetrazolium salt to formazan using the WST-1 assay kit (Roche) according to the manufacturer’s protocol. THP-1 monocytes were seeded in 48-well plates (4.6 × 10^5^ cells per well) and differentiated into macrophages. Macrophages were infected with *B. pertussis* cells at different MOIs as described above. Briefly, after 1 h of co-incubation, nonadherent bacteria were removed by washing, and remaining extracellular bacteria were killed by incubation in RPMI medium containing 100 μg/ml of polymyxin B sulphate (Sigma) for 1 h. At this time point, corresponding to 2 h pi, infected cells were washed intensely with prewarmed RPMI medium and then incubated in 200 µl of RPMI and 20 µl of WST-1 substrate for 40 min at 37°C. In parallel, uninfected cells were treated in the same manner and served as controls (100% viability). The absorbance of formazan dye measured at 450 nm correlates directly with the number of viable cells and was measured with a scanning multi-well spectrophotometer Epoch (BioTek).

Cytotoxicity toward THP-1 cells was determined using the CellTox™ Green Cytotoxicity Assay (Promega), which measures changes in membrane integrity that occur as a result of cell death. The fluorescent signal generated by binding of the dye to the dead-cell DNA is proportional to cytotoxicity. THP-1 monocytes grown in colorless RPMI medium (R7509, Sigma) supplemented with L-glutamine (0.03%) were seeded in 96-well plates (1 × 10^5^ cells per well) and differentiated into macrophages. The differentiated macrophages were infected by adding 100 µl of a *B. pertussis* cell suspension (containing 5 × 10^6^ cells; MOI 50). After 30 min of co-incubation, nonadherent bacteria were removed by washing, and the remaining extracellular bacteria were killed by incubation in RPMI medium containing 100 μg/ml of polymyxin B sulphate (Sigma) for 30 min. At this time, the infected cells were washed intensely with prewarmed RPMI medium, and the assay was started by adding of CellTox^TM^ reagent according to the manufacturer’s protocol. Uninfected cells were treated in the same manner and served as controls. Next, the macrophages were incubated in a Tecan Spark multimode microplate reader (37°C, 5% CO2), and the fluorescent signal was measured at Ex/Em = 490 ± 10/520 ± 10 nm for 12 h after addition of reagent.

## Results

### Parallel transcriptomic profiling of infected macrophages

Monocyte-derived THP-1 macrophages were infected in triplicate with 4.0 × 10^7^ cells per well of the *B. pertussis* reference strain Tohama I. Samples of infected macrophages were collected at 1, 2, 4, 8, and 12 h (time points T1–T5) post-infection (pi). Control samples (C1–C5) of uninfected macrophages and intact bacteria cultivated in triplicate in RPMI medium were incubated and harvested simultaneously. In parallel with RNA isolation, plating of lysed infected macrophages revealed that the macrophages contained approximately 4.1 × 10^6^, 4.8 × 10^6^, 4.0 × 10^6^, 4.4 × 10^6^, and 3.3 × 10^6^ viable bacteria per well at time points T1, T2, T3, T4, and T5, respectively.

**Figure 1.**
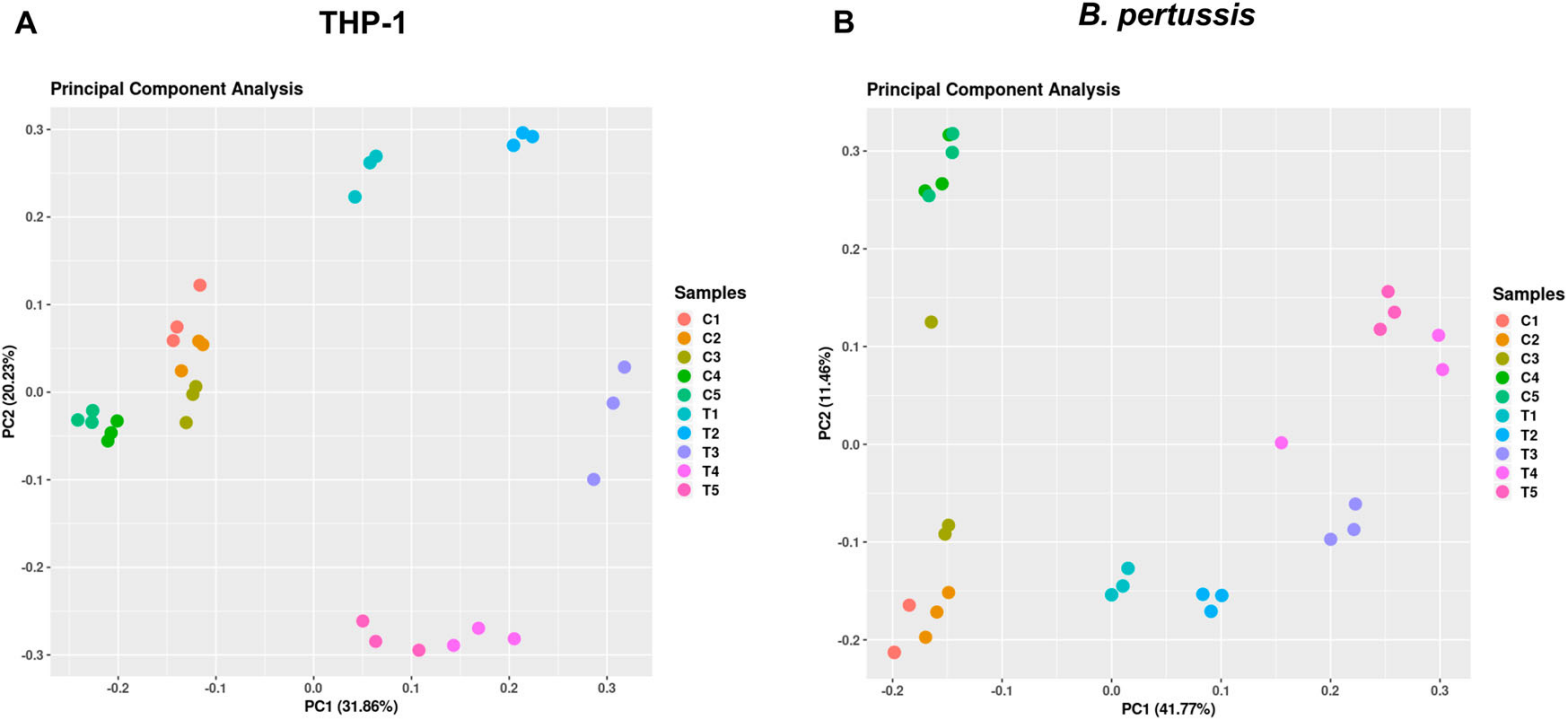
(a) Principal component analysis was performed with transcriptomic profiles of uninfected (C1-C5) and infected (T1–T5) THP-1 macrophages harvested 1 h (T1), 2 h (T2), 4 h (T3), 8 h (T4), and 12 h (T5) pi. (b) Principal component analysis was performed with transcriptomic profiles of control *B. pertussis* cells incubated in RPMI medium (C1–C5) and intracellular *B. pertussis* cells (T1–T5) harvested 1 h (T1), 2 h (T2), 4 h (T3), 8 h (T4), and 12 h (T5) pi. Each dot in both panels represents an independent biological replicate.

RNA-seq analysis of samples isolated from infected cells yielded between 50 and 120 million reads, whereas control samples (uninfected macrophages and bacteria incubated in RPMI medium) yielded an average of 20 million reads (Figure S1). Reads were mapped to human and B. *pertussis* genomes and all samples were clustered by principal component analysis (PCA). PCA revealed that samples of infected macrophages clustered separately from uninfected cells (Figure 1(a)). Similarly, samples of intracellular bacteria clustered apart of control samples along principal component 1 (PC1), which accounted for nearly 42% of the observed variance (Figure 1(b)). The control samples also exhibited some degree of variability along PC2 (≈ 12%), although the samples of intracellular bacteria displayed a similar trend toward PC2, but to a lesser extent. Therefore, we cannot exclude the possibility that some variance in the control samples was due to RPMI medium-specific effects. Importantly, samples of both infected macrophages and intracellular bacteria collected at each time point clustered separately, indicating significant time-dependent changes in gene expression profiles during infection in both the host and the pathogen as early as 1 h pi.

### Global changes in transcriptional profiles of internalized *B. pertussis* cells

Next, we focused on samples of *B. pertussis* to monitor temporal changes in gene expression profiles of intracellular bacteria during the course of infection. To this end, we performed pairwise differential expression (DE) analysis to compare the transcriptomic profiles of intracellular and control bacteria at each of the five time points. The DE analysis identified 455, 708, 1112, 1255, and 1243 significantly modulated ($|log_{2}FC|\geq 1$ ; adjusted *p*-value < 0.05) genes at time points T1 to T5, respectively (Table S1). Similarly, 86, 137, 160, 130, and 110 non-coding RNAs, including rRNAs, tRNAs, and previously identified candidate non-coding RNAs [45], were significantly modulated at the corresponding time points. Detailed analysis of protein-coding genes revealed that 1368 genes were modulated at least at two time points and 148 genes were modulated at all five time points (Figure S2). To validate the RNA-seq data, we analyzed the expression profiles of randomly selected significantly modulated genes by quantitative PCR. This experiment revealed that, in agreement with the RNA-seq data, the expression of the virulence genes *fhaB*, *prn*, *vag8*, *bsp22*, *brkA*, and *tcfA* was decreased in internalized cells and the expression of *BP2871*, *vrg6* and *vrg24* genes was increased (Figure S3).

**Figure 2.**
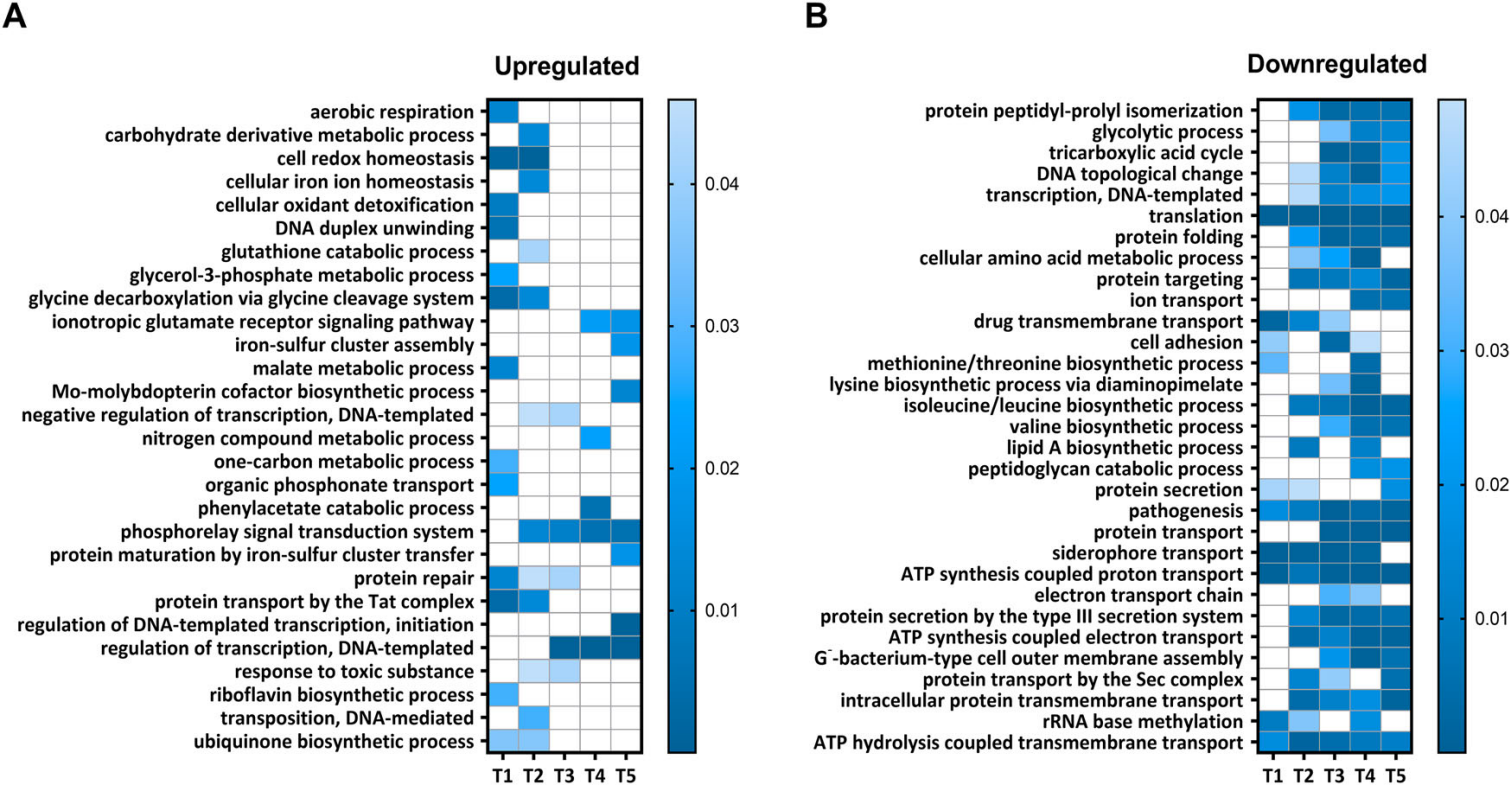
GO enrichment analysis of processes modulated in intracellular *B. pertussis* cells. Analysis was applied to identify GO terms from the domain “Biological processes” enriched for either upregulated (panel a; log_2_FC > 0) or downregulated (panel b; log_2_FC < 0) DE genes at all experimental time points (T1–T5). The processes that were significantly enriched at least at one time point were selected for visualization. The vertical bars show the adjusted *p*-values (shades of blue) for both sets of genes.

To gain deeper insight into the functional clustering of upregulated and downregulated genes at each time point, we performed gene ontology (GO) term enrichment analyzes. This analysis focused on biological processes and revealed the GO terms enriched for genes that displayed either increased or decreased expression during the intramacrophage phase (Figure 2). Among the biological processes enriched in upregulated genes, we identified those related to the “Phosphorelay signal transduction system,” “Regulation of transcription,” “Cell redox homeostasis,” and “Protein repair” (Figure 2(a)). On the other hand, processes such as “Pathogenesis,” “Siderophore transport,” “Intracellular protein membrane transport” and several processes linked to the energy metabolism and biosynthesis of amino acids were significantly enriched in downregulated genes (Figure 2(b)).

### Adaptation of *B. pertussis* to the intramacrophage environment

**Figure 3.**
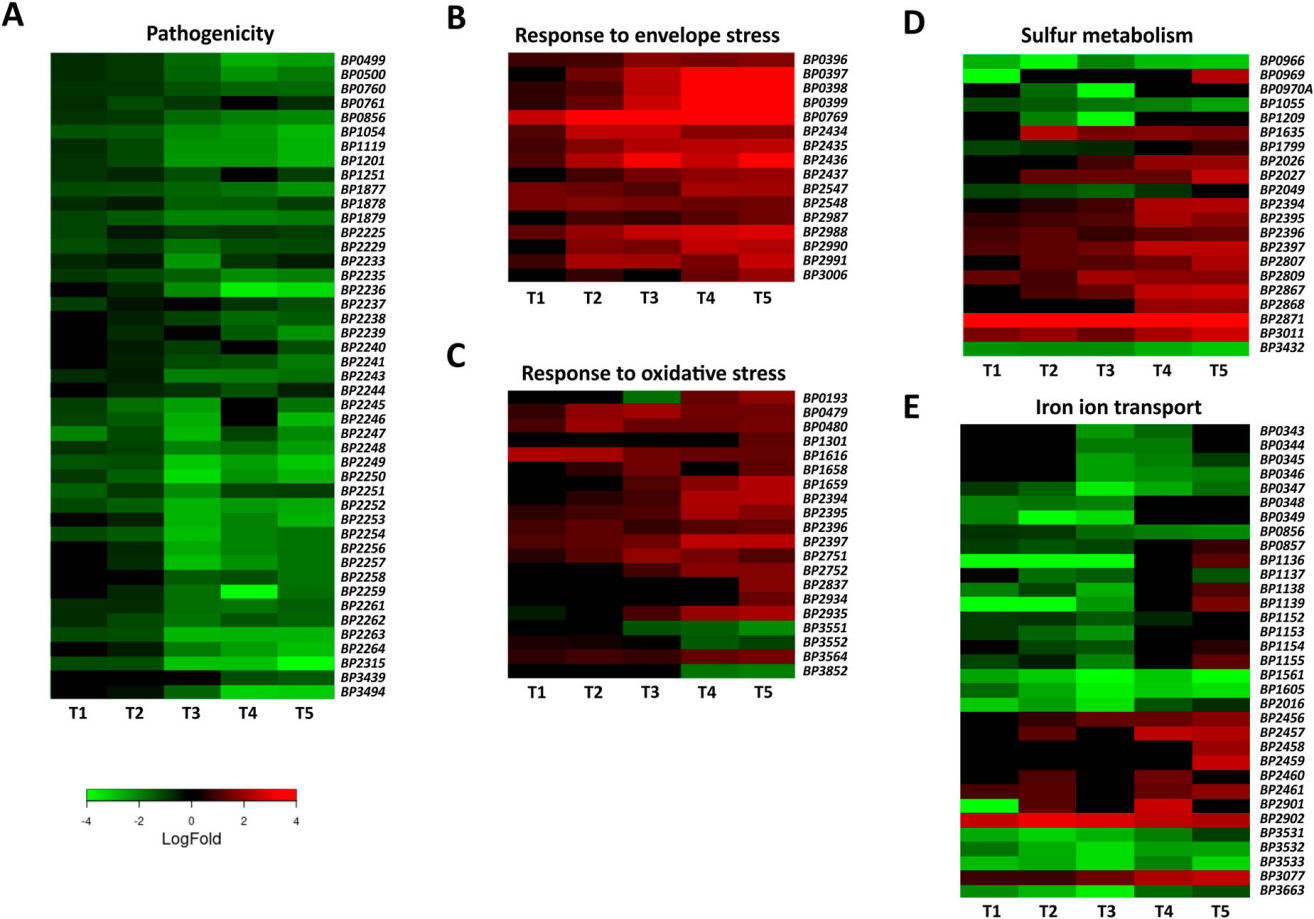
Adaptation of *B. pertussis* to the intramacrophage environment. Heat maps depicting log_2_ fold changes in the expression of *B. pertussis* genes (intracellular/intact bacteria) during infection (T1–T5). Shown are genes involved in pathogenesis (a), response to membrane stress (b), response to oxidative stress (c), sulphur metabolism (d), and iron transport (e).

GO enrichment analysis revealed that several important biological processes are enriched in significantly modulated genes. Therefore, we examined these processes in more detail (Figure 3).

#### Pathogenicity

Consistent with our previous report [23], the expression of majority of virulence factors was significantly reduced within macrophages. As can be seen from Figure 3(a), this effect occurred mainly after 2 h pi and was due to reduced expression of the *bvgA* (*BP1877*) and *bvgS* (*BP1878*) genes, the main regulators of virulence. Among the downregulated genes, we identified those encoding adenylate cyclase toxin (*BP0760*), dermonecrotic toxin (*BP3439*), adhesins such as filamentous haemagglutinin (*BP1879*), serotype 2 fimbriae (*BP1119*), and pertactin (*BP1054*), autotransporters Vag8 (*BP2315*) and BrkA (*BP3494*), tracheal colonization factor A (*BP1201*), and the nearly complete *bsc* locus encoding the type 3 secretion system (*BP2225–BP2264*).

#### Membrane composition and envelope stress

Internalized bacteria are subjected to various stresses resulting from harsh conditions within macrophages. Low pH, and the activity of reactive oxide species and antimicrobial peptides exert strong effects on cell wall integrity. Our results indicate that intracellular *B. pertussis* cells actively adapted their membranes to macrophage-induced stress, as the expression of several genes and operons involved in fortifying the cell wall was increased, especially at later time points (Figure 3(b)). For example, the expression of the *BP0396–0399* cluster, which affects LPS composition and lipid A remodelling, was strongly upregulated. Similarly, the *BP0769* gene, encoding the d-amino acid transferase required for the synthesis of the peptidoglycan components d-alanine and d-glutamate, and the *dra* locus (*BP2987–BP2991*), required for the incorporation of d-alanine into outer membrane, were incessantly upregulated during infection. In addition, the locus *BP2434–BP2437*, which contains the *mucD*, *mucB*, *rseA*, and *rpoE* genes that encode factors involved in the response to outer membrane stress, was highly overexpressed. The expression of the lipid A palmitoyltransferase gene *pagP* (*BP3006*) was increased at later time points. On the other hand, the expression of porin genes such as *BP0840*, *BP0943* (*ompA)*, *BP3405* (*ompQ*), and *BP3755* (*ompW*), which enable the transport of small molecules including antimicrobial peptides, was downregulated (Table S1). The expression of *lpx* (*BP1429–BP1432*) and *bpl* (*BP0089–BP0093*) operons, which are responsible for lipooligosaccharide biosynthesis, was also significantly decreased (Table S1).

#### Oxidative stress and redox homeostasis

During infection, the production of reactive oxygen species resulting from bacterial metabolism and from the activity of immune cells can substantially damage bacterial macromolecules. Our data suggest that intracellular *B. pertussis* cells responded to oxidative stress (Figure 3(c)). Indeed, the expression of the *dps* (*BP1616*) gene encoding the DNA-binding protein that confers resistance to hydrogen peroxide by absorbing the oxidative damage in place of the DNA [46] was increased throughout the experiment. The expression of the redox-sensitive regulator gene *soxR* (*BP2837*) and the Mn-dependent superoxide dismutase *sodA* (*BP0193*) was significantly increased, though only at later time points. In addition, the expression of paraquat-inducible genes *pqiA* (*BP2751*) and *pqiB* (*BP2752*) and glutathione peroxidase gene (*BP1301*) was also increased during infection. Moreover, our analysis revealed several highly expressed genes involved in the repair of damaged proteins. Indeed, the sulphur-containing amino acids methionine and cysteine are particularly sensitive to oxidative stress because of their electron-rich sulphur atom. During infection, genes encoding cytosolic MsrB (*BP3564*) and periplasmic MsrP (*BP0479*) and MsrQ (*BP0480*) methionine reductases were significantly upregulated. Our data also showed that the locus encoding the putative ATP-binding cassette transporter (*BP2394–BP2397*) with specificity for di- and tripeptides (*e.g*. glutathione) was strongly overexpressed during the course of infection. In contrast, the entire *pha* locus (*BP1691*–*BP1696*), which encodes the potassium efflux system involved in pH adjustment, was downregulated (Table S1).

#### Sulphur metabolism

Transcriptomic analysis revealed that the expression of a variety of genes controlling sulphur metabolism was strongly modulated (Figure 3(d)). The reductive sulphate assimilation pathway, which is required for sulphate transport and cysteine biosynthesis, was attenuated during infection. In particular, the *sbp* (*BP0966*), *cysD* (*BP0970*), *cysG* (*BP1055*), *cysJ* (*BP1209*), sulphate permease *BP2049*, *cysI* (*BP3432*), and adenosylhomocysteinase *BP3068* genes were significantly downregulated. The *BP2816–BP2818* locus, which encodes the putative methionine importer, was also downregulated. On the other hand, the expression of genes involved in cysteine export and catabolism was strongly increased. For example, the genes *BP2026* and *BP2027* genes, encoding a probable cysteine exporter and a sulphite exporter, respectively, and the two putative cysteine dioxygenase genes *BP2871* and *BP3011* were strongly upregulated.

#### Metal homeostasis

During infection, bacterial pathogens enter a hostile and variable environment that contains limited amounts of essential nutrients, including metals. Therefore, we were surprised to see that several iron transport genes and systems were downregulated during infection, predominantly within the first 4 h pi (Figure 3(e)). For example, the expression of the *fecI* and *fecR* genes, which control the transport of ferric citrate, and the *ftrABCD* operon (*BP1152*–*BP1155*), which facilitates the transport of ferrous iron, was strongly downregulated within the first four hours after infection. Similarly, expression of the haem transport system encoded by the *bhuRSTUV* locus (*BP0343*–*BP0347*) and adjacent regulatory genes *hurR* (*BP0348*) and *hurI* (*BP0349*) was significantly downregulated during infection. The expression of *BP3531–BP3533* cluster, which consists of the *tonB*, *exbB*, and *exbD* genes involved in iron binding and transport, was also strongly reduced. In contrast, the expression of the *alc* operon (*BP2456*–*BP2461*), which is responsible for the biosynthesis of alcaligin siderophores, increased significantly 8 h after infection. Genes responsible for the binding (*BP2901*) and release (*BP2902*) of enterobactin siderophores were also overexpressed.

The expression of several genes involved in the homeostasis of other essential metals was modulated as well. For example, the magnesium importer gene *mgtC* (*BP0414*), the putative manganese importer locus *BP3080–82*, and the putative nickel/cobalt transporter gene *BP3078* were strongly upregulated (Table S1).

#### Central and energy metabolism

Our data indicate that intracellular cells extensively rewired their metabolic activity and energy production upon entry into host macrophages (Table S1). Most strikingly, the large *nuo*, *sdh* and *atp* operons, encoding the electron transfer chain complexes of NADH:ubiquinone oxidoreductase, succinate dehydrogenase, and ATP synthase, respectively, were strongly downregulated from the beginning of infection. Furthermore, expression of the *cyo* and *cyd* operons encoding terminal ubiquinol oxidases and genes encoding terminal cytochrome cbb3 oxidase were also significantly downregulated.

In addition, several important metabolic processes were substantially modulated in internalized bacteria (Table S1). Among others, several genes involved in glycolysis, such as *gap*, *pgk*, and *ldh*, were downregulated. On the other hand, the expression of *glcDEF* genes involved in glycolate/glyoxylate metabolism (*BP2903*–*BP2905*) and the glyoxalase gene *BP2863* was strongly upregulated. Similarly, the expression of genes responsible for the biosynthesis (acetolactate synthase *BP0467*) and transport (ABC transporters *BP0619–BP0622* and *BP3573–BP3577*) of branched-chain amino acids, required for the metabolism of fatty acids (acetyl-CoA synthetase *BP0661*, enoyl-CoA hydratases *BP0627* and *BP0662*, and long-chain fatty acid ligase *fadD*), and for phenylacetic acid catabolic pathway (*BP2675–BP2684*), was significantly increased.

### Large part of the BvgAS regulon is modulated in internalized cells

**Figure 4.**
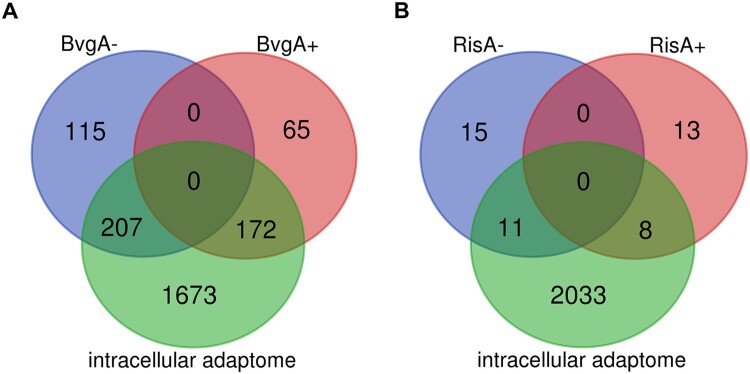
Comparison of the intracellular adaptome with BvgAS and RisAK regulons. (a) Venn diagrams showing the number of genes differentially expressed in intracellular *B. pertussis* cells at least at one time point (intracellular adaptome), the number of *B. pertussis* genes repressed (BvgA−) or activated (BvgA+) by the BvgAS system (reference [9] in the manuscript), and the overlap between each dataset. (b) Venn diagrams showing the number of genes differentially expressed in intracellular *B. pertussis* cells at least at one time point (intracellular adaptome), the number of *B. pertussis* genes repressed (RisA−) or activated (RisA+) by the RisAK system (reference [48] in the manuscript), and the overlap between each dataset.

The greatly reduced expression of virulence genes suggested that intramacrophage environment induces an avirulent phenotype in internalized *B. pertussis* cells. We noticed that in addition to a large number of virulence genes, a great variety of other genes belonging to the BvgAS regulon were identified among the DE genes. Therefore, we determined the extent to which the BvgAS regulon overlaps with the set of genes found in our DE analysis which we named “intracellular adaptome”. In this analysis, we considered all *bvg*-dependent genes published by Moon et al. [47]. The rationale behind this was that when internalized cells develop the Bvg^−^ phase, then all genes that are downregulated by the BvgAS system (virulence-repressed genes) should be upregulated and *vice versa*, genes that are activated by the BvgAS system (virulence-activated genes), should be downregulated upon phagocytosis. Interestingly, 207 of 322 virulence-repressed genes (64.2%) were significantly upregulated at least at one time point in internalized cells and 172 of 237 virulence-activated genes (72.5%) were significantly downregulated in intracellular bacteria (Figure 4(a)). A large proportion of the DE genes do not overlap with the BvgAS regulon.

Expression of at least some of the virulence-repressed genes in *B. pertussis* is induced by the RisAK two-component system [48]. Therefore, we compared the intracellular adaptome with the list of genes shown to be transcriptionally regulated by the RisA factor (Figure 4(b)). Analogously to BvgA, we determined whether genes activated by RisA were upregulated and genes repressed by RisA were downregulated in intracellular bacteria. This analysis showed that among the genes activated by RisA, 8 of 21 (38.0%) were upregulated, including *vrg6* and several genes encoding putative lipoproteins and small membrane proteins. Of the genes repressed by RisA, 11 of 26 (42.3%) were downregulated in intracellular bacteria, including several flagellar genes. Similar to BvgA, a large part of the DE genes do not overlap with the RisA regulon.

### Cysteine dioxygenases impact on pathogenesis of *B. pertussis*

**Figure 5.**
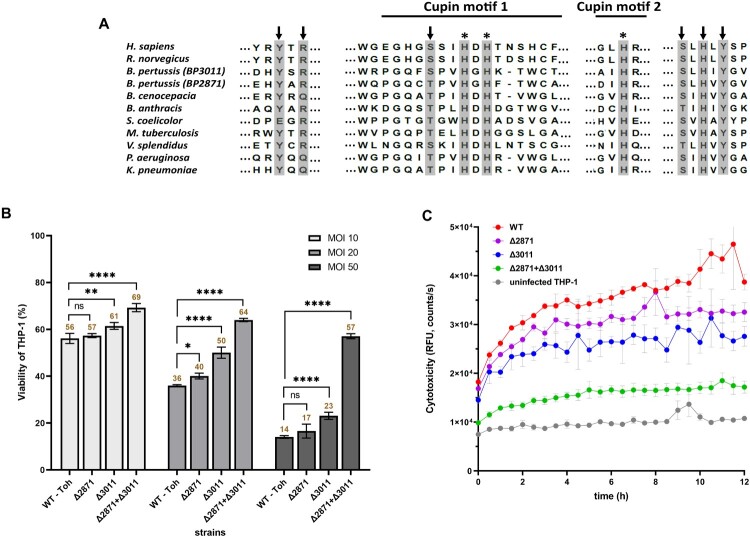
Impact of *cdo* genes on *B. pertussis* pathogenicity. (a) Conservation of functional residues in CDO enzymes between *B. pertussis* and other organisms. The grey rectangles show important residues that are either part of the CDO active site (arrows) or involved in iron binding (asterisks). The extent of cupin motifs 1 and 2 is depicted with solid lines. Uniprot query numbers are as follows: *Homo sapiens*, (Q16878); *Rattus norvegicus*, (P21816); *Bordetella pertussis BP3011*, (Q7VUR5); *Bordetella pertussis BP2871*, (Q7VV35); *Burkholderia cenocepacia*, (A0A142PCQ1); *Bacillus anthracis*, (A0A1T3V1L5); *Streptomyces coelicolor*, (A0A6M9XRZ5); *Mycobacterium tuberculosis*, (A0A045IN01); *Vibrio splendidus*, (A0A0P6ZFX5); *Pseudomonas aeruginosa*, (A0A0F7QLY4); *Klebsiella pneumoniae* (A0A086IHW3). (b) Viability of THP-1 macrophages infected with *cdo* mutants of *B. pertussis*. Macrophages were infected in triplicate with the *B. pertussis* wt strain and Δ*BP2871*, Δ*BP3011*, and Δ*BP2871*Δ*BP3011* mutants at MOIs of 10, 20, and 50 bacteria per macrophage and incubated for 2 h at 37°C. Next, macrophages were washed with fresh RPMI and finally incubated in 200 μl RPMI and 20 μl WST-1 reagent for 40 min. After incubation, the absorbance of the samples, which is proportional to cell viability, was measured at 450 nm using a multi-well spectrophotometer. The absorbance of uninfected cells treated in parallel in the same manner was arbitrarily set to 100%. The bars represent mean values ± standard deviation, the labels above the bars indicate the mean values of cell viability (%). Statistical analysis was performed using a two-way ANOVA test for multiple comparisons (Dunnett’s test); ns, *p*-value > 0.05, *, *p*-value < 0.05, **, *p*-value < 0.005, ****, *p*-value < 0.00005. The result is representative of three independent experiments. (c) Cytotoxicity of *cdo* mutants of *B. pertussis* toward THP-1 macrophages. Macrophages were infected in triplicate with the *B. pertussis* wt strain and the Δ*BP2871*, Δ*BP3011*, and Δ*BP2871*Δ*BP3011* mutants (MOI of 50). Uninfected cells served as controls. Immediately after addition of the fluorescent dye, THP-1 cells were incubated for 12 h (37°C, 5% CO_2_) in the microplate reader. During incubation, the fluorescence of the samples, which is proportional to cytotoxicity, was measured every 30 min. The graph shows mean values and the standard errors of the means. The result is representative of two independent experiments.

DE analysis revealed that a large number of genes responsible for the transport and metabolism of cysteine were modulated in intracellular *B. pertussis* cells. Among them, two putative cysteine dioxygenases (CDO) genes, *BP2871* and *BP3011*, were highly upregulated upon internalization, and *BP2871* in particular was among the most deregulated genes in the DE analysis (log_2_FC > 7). Consistent with their annotation, alignment of the two enzyme sequences with the Uniprot database identified several domains characteristic of the CDO protein family (Figure 5(a)). Given the extent of upregulation, we wondered whether the *cdo* genes play a significant role in intracellular *B. pertussis* cells. Therefore, macrophages were infected at three different multiplicity of infection (MOI) doses of 10, 20, and 50 bacteria per macrophage and viability was tested 2 h pi (Figure 5(b)). First, we tested a single Δ*BP2871* mutant and determined its cytotoxicity, *i.e.* its capacity to reduce the viability of THP-1 macrophages, however, we found no significant difference compared to wt strain. The viability of macrophages infected with the Δ*BP3011* mutant lacking the other *cdo* gene was slightly but significantly increased at all MOIs, however, the viability of infected cells was still substantially reduced compared with uninfected macrophages, especially at the highest MOI. We hypothesized that the two *cdo* genes might complement each other and therefore, we constructed a double Δ*BP2871*Δ*BP3011* mutant lacking both *cdo* genes. The viability of THP-1 cells infected with the double mutant was significantly higher than that of cells infected with the wt strain and both single *cdo* mutants at all MOIs tested (Figure 5(b)). To confirm these results, we examined the cytotoxic effects of all strains toward macrophages in real time using an assay that measures changes in membrane integrity resulting from cell death (Figure 5(c)). Consistent with the cell viability assays, the cytotoxic effects toward THP-1 cells caused by the double mutant were strongly reduced compared with the wt strain and the two single *cdo* mutants (Figure 5(c)).

## Discussion

Although *B. pertussis* is classified as an extracellular pathogen, it has been reported that these bacteria can also survive and persist within host cells, including both professional and nonprofessional phagocytes [20,49]. It has been hypothesized that the ability for intracellular persistence is conserved among mammalian pathogens of *Bordetella* species and that the intracellular phase contributes to infection [26,27,50]. In this regard, our data indicate that after the initial elimination of extracellular bacteria, viable intracellular *B. pertussis* cells repopulate the antibiotic-free RPMI medium during infection. Approximately 10^4^ *B. pertussis* cells per well (≈ 1% of intracellular bacteria) are found in the extramacrophage environment (data not shown), a number very similar to that of *B. pertussis* cells released from human epithelial A549 cells [49]. This observation suggests that bacteria that survive in macrophages may contribute to disease transmission.

The conservation of intracellular persistence would imply that *Bordetella* species have evolved a specific expression programme to adapt to the intramacrophage environment. Therefore, we have employed time-resolved RNA-seq approach to specifically monitor and describe the adaptation of *B. pertussis* to the intramacrophage environment to elucidate the capacity of this reemerging pathogen to persist within this niche. Our data clearly demonstrate that *B. pertussis* cells actively adapt to the harsh environment within macrophages early after internalization. The changes in global gene expression profiles were enormous, with more than 30% of genes differentially expressed at least at two time points during the course of infection. Nevertheless, we are aware that part of the observed effect could be due to RPMI-specific effects on the expression of control bacteria, especially at the last two time points. In our pilot experiment we identified 310 bacterial genes that were induced 6 h post-infection [23]. Compared to here presented study, 79.3% of these genes displayed identical expression patterns (data not shown). In addition, we compared the results of our study with proteomic analysis of intracellular *B. pertussis* cells isolated from THP-1 macrophages. This study differed in some important details (*e.g*. MOI) that could affect the dynamics of infection and gene/protein expression, nevertheless, of the 203 proteins modulated 3 h pi we found 82 genes (40%) that displayed similar trends in expression/production.

Remarkably, this global rewiring of gene expression profiles in intracellular cells was closely associated with the switch from the Bvg^+^ to Bvg^−^ phase. Yet, the magnitude of the “intracellular adaptome” exceeded more than twice the extent reported for the BvgAS regulon [9], suggesting that the adaptation of *B. pertussis* to the intramacrophage environment is orchestrated also by additional regulators. Of note, 100% of *B. pertussis* colonies obtained by direct plating of lysed macrophages (containing viable intracellular bacteria) produced adenylate cyclase toxin, as demonstrated by clear zones on BG agar supplemented with blood (data not shown). This result suggests that the BvgAS system remained functional and was only temporarily silenced in macrophages.

In agreement with previous observations [23,26], our data indicated that the transcriptomic profiles of intracellular *Bordetella* cells differ substantially from those of bacteria isolated from the respiratory tract of mice [30,31,43]. Hallmark of this difference, similar to *B. bronchiseptica* [26], was the expression of virulence factors, which was strongly downregulated in intracellular cells. After host infection, extracellular bacteria express virulence genes to successfully colonize epithelial layers and manipulate immune cells to evade phagocytic clearance. Most likely, these “contact weapons” such as adhesins, toxins, autotransporters, and T3SS are required for colonization and immune system evasion in the host tissues, but are not required after internalization. We presume that intracellular *B. pertussis* cells have evolved an alternative strategy which appears to be based in part on a rapid shift from virulent to avirulent mode resulting from downregulation of the *bvgAS* two-component system, a key regulator of *Bordetella* virulence. Our data indicate that activation of the Bvg^−^ phase augments a very complex adaptive process based on adaptation of central and energy metabolism, fortification of the cell wall, maintenance of appropriate redox and metal homeostasis, and repair of damaged macromolecules.

Internalized *B. pertussis* cells are exposed to various stress factors such as reactive oxidative species, nutrient limitation, antimicrobial peptides, and low pH. Our results indicate that the adaptation of intracellular *B. pertussis* cells is multifaceted to integrate the response to multiple stressors. Maintenance of the cell envelope is one of these crucial processes, and our results suggest that *B. pertussis* copes with outer and inner membrane stresses induced by a hostile environment. For example, several genes that have been shown to be involved in membrane integrity protection (*pqiA* and *pqiB*) [51] or resistance to hydrogen peroxide (*dps*, glutathione peroxidase gene) [46,52] were upregulated. We also noticed that as a common theme, the mechanisms controlling cell envelope modifications were strongly upregulated, whereas the processes leading to *de novo* synthesis of the cell wall components were attenuated. Indeed, several factors involved in the modification of already transported lipooligosaccharide and peptidoglycan required for cell wall integrity of bacterial pathogens were strongly overexpressed. For example, lipid A modifications by glucosamine and palmitate [53,54] and peptidoglycan or outer membrane modification by d-alanine [55,56] have been shown to be required for resistance to antimicrobial peptides. In contrast, genes involved in the biosynthesis of lipid A and lipooligosaccharide were attenuated. We also observed downregulation of the *nuo*, *cyo* and *atp* operons, which encode large respiratory protein complexes in the cytoplasmic membrane. Remarkably, downregulation of these complexes has also been observed in bacteria responding to membrane stress by activation of the two-component regulatory system CpxAR [57]. Indeed, stressors such as low pH and the presence of misfolded proteins trigger the Cpx stress response, which mediates adaptation to cell envelope stresses by downregulation of nonessential protein trafficking between the cytosol and the inner membrane [58]. Although we did not identify plausible homologs of *cpxAR* genes in *B. pertussis*, we speculate that the observed reduced *de novo* biosynthesis of membrane components and decreased expression of large membrane complexes in intracellular *B. pertussis* cells also contributes to the maintenance of envelope integrity. Of note, the expression of several genes encoding outer membrane proteins was decreased. A similar pattern was observed in *Salmonella* and reduced outer membrane permeability was found to provide protection against toxic molecules [59]. Furthermore, to escape the antimicrobial effects of acidic pH, *B. pertussis* cells modulated the expression of several genes involved in the maintenance of pH homeostasis. For example, in agreement with a previous report [60], the expression of the magnesium importer *mgtC* homolog (*BP0414*) was increased during infection. MgtC has been shown to be an important adaptive factor for intramacrophage survival of bacterial pathogens [61], as it inhibits ATP synthase activity and consequently, hinders the proton influx and acidification of the environment [62]. Also, the strong repression of the *pha* locus, which encodes the potassium efflux pump, may have contributed to the neutralization of the intramacrophage milieu as the accumulation of potassium has been shown to be essential for pH homeostasis under acidic conditions [63].

The phagosome represents a hostile, nutrient-poor environment, and therefore, intracellular pathogens must adjust their metabolic activities. Our study revealed that internalized *B. pertussis* cells have extensively remodelled their metabolism to take advantage of the nutrients available within the phagosome. Importantly, most of the upregulated metabolic processes were facilitated by genes previously shown to be repressed by the BvgAS system [9]. This metabolic shift was highlighted by the induction of genes involved in the β-oxidation of fatty acids and the glycolate/glyoxylate pathway, which allow cells to utilize fatty acids-derived metabolites and convert them into simple carbon molecules [64]. These genes have also been upregulated in intracellular *B. bronchiseptica* cells [26], and glyoxylate production has been shown to be important for intramacrophage survival of *M. tuberculosis* [65]. In addition, genes associated with the tricarboxylic acid cycle, lipid metabolism, and branched amino acid transport were strongly upregulated. Of note, gene encoding glyoxalase, an enzyme that detoxifies methylglyoxal, was also intensely induced. Methylglyoxal is a reactive electrophilic byproduct of metabolism and its removal has been reported to contribute to the survival of the intracellular pathogen *Listeria monocytogenes* [66]. The remodelling of metabolic fluxes in intracellular cells was also illustrated by extensive changes in the expression of *Bordetella* uptake genes (bugs). This group of genes encodes periplasmic solute-binding proteins that bind and deliver their specific cargo, such as amino acids and citrate, to their cognate membrane components [67,68]. *B. pertussis* contains more than 70 Bug proteins [69], and we found that about 73% of them were significantly modulated at least at one time point during infection.

One of the most interesting observations in our study was the large number of strongly and significantly modulated genes required for sulphur metabolism. Among the significantly downregulated genes were those involved in the sulphate assimilation pathway. Intriguingly, speciation of *B. pertussis* was accompanied by the loss of function of several genes required for sulphate uptake, such as *cysT*, *cysW*, and *cysH* [70]. Furthermore, our data indicate that most of the functional genes within the *cys* operon and genes involved in the biosynthesis and transport of sulphur-containing compounds such as cysteine and methionine were downregulated. Apparently, intracellular *B. pertussis* cells diminished the biosynthesis and uptake of cysteine and other sulphur-containing compounds in several steps. Cysteine is an important amino acid and nutrient, however, due to its highly reactive thiol group, elevated cytosolic cysteine levels are toxic [71]. Cysteine homeostasis is therefore maintained by several mechanisms including metabolic redistribution to non-reactive thiols (*e.g.* glutathione), cysteine efflux, and cysteine degradation, all of which ensure the removal of excess cysteine [72,73]. This is likely also true for *B. pertussis*, as several genes required for cysteine catabolism and export have been strongly upregulated including the cysteine exporter and two CDOs. The biology and function of CDO enzymes are best studied in eukaryotic cells [74], yet, CDOs have also been identified in bacterial cells [73]. These enzymes, which belong to the cupin family of proteins, play an important role in cysteine homeostasis by initiating the conversion of cysteine into beneficial products such as pyruvate [73,74]. The human pathogenic yeast *Candida albicans* lacking the CDO gene induced delayed mortality in a mouse model [75]. Our experiments revealed that the cytotoxicity of the *B. pertussis* double CDO mutant toward human macrophages was strongly reduced and thereby highlighted the importance of cysteine homeostasis for *B. pertussis* adaptation and survival. Though we provide only preliminary evidence, we speculate that CDO activity plays an important role in the physiological fitness of other bacterial pathogens.

The intramacrophage environment is deprived of essential metals, and therefore pathogens need to scavenge metals using high-affinity acquisition systems. After infection, the expression of iron utilization systems of *B. pertussis* was differentially phased within macrophages. A similar phenomenon was observed in *B. pertussis* cells during colonization of the mouse respiratory tract [76]. Especially within the first 4 h pi, several iron transport systems and iron starvation-inducible genes were downregulated, suggesting that intracellular *B. pertussis* cells were not limited by iron during the early phase of infection. On the other hand, expression of loci involved in the utilization of enterobactin and alcaligin siderophore systems was increased at later time points. These results suggest that siderophores became an essential and preferential system for iron acquisition in intracellular bacteria during the course of infection. We hypothesize that *B. pertussis* cells adapt to the iron resources available within the intracellular niche (lack of free iron) and use the siderophores to acquire iron from host iron-loaded molecules such as ferritin.

Remarkably, expression of the *BP3077*–*BP3082* operon increased strongly and steadily during infection. This operon encodes a TonB-dependent receptor (*BP3077*), a putative nickel/cobalt transporter (*BP3078*), a DUF1007 family protein (*BP3079*), and an ABC transporter (*BP3080*–*BP3082*) that shares a substantial homology with the manganese importer SitABCD of *Salmonella enterica* subsp. *Typhimurium* [77]. We have previously shown that, as an evolutionary adaptation to “nutritional immunity” evolved by immune cells [78], *B. pertussis* has decayed its manganese exporter to accumulate this important metal within the cytosol, thereby enhancing its survival under oxidative stress [79]. Furthermore, a neighbouring *BP3083* gene transcribed opposite to this operon encodes a Fur family transcriptional regulator. In contrast to adjacent operon, the expression of the regulatory gene was not affected upon internalization. It will be of utmost importance to decipher the role of this locus in the adaptation of *B. pertussis* to the intramacrophage environment.

Transcriptomic analysis also revealed several significantly modulated non-coding RNAs previously identified in *B. pertussis* [45]. Among the strongly downregulated candidate transcripts, we identified CT_433 and CT_233, whereas the expression of CT_347, CT_481, CT_483, and CT_531 was significantly increased during infection (see Table S1). Of particular note, the CT_433 transcript was recently identified also by bioinformatics (referred to as the S17 transcript) and subsequent analyzes showed that this sRNA represents a Bvg-activated gene [47]. In support, expression of the CT _433 sRNA gene was among the most downregulated genes in our analysis (log_2_FC < −10), providing further evidence that the intramacrophage environment is inducible for Bvg^−^ mode.

Importantly, our results may well explain the preservation of the seemingly superfluous Bvg^−^ phase in the infection cycle of *B. pertussis*. The Bvg system in the closely related mammalian pathogen *B. bronchiseptica* has been shown to contribute to survival in nutrient-poor environments outside the host, as well as to intracellular survival and replication within amoebae [24,80]. *B. pertussis* represents a strictly human pathogen that does not survive outside its host, and thus it has been hypothesized that the Bvg^−^ phase represents an evolutionary remnant that is otherwise useless in the infection cycle of this pathogen [8,10]. Alternatively, it has been speculated that the avirulent phase may play a role in either the transmission of the disease (aerosol route) associated with the temperature drop [9,81] or the survival and persistence of the bacterium in the host [27]. Regarding the first alternative, we have shown that, unlike *B. bronchiseptica*, the activity of the *bvg* system in *B. pertussis* is only partially reduced at low temperatures, allowing the production of virulence factors at temperatures below 25°C [82]. We hypothesized that *B. pertussis* cells do not switch to Bvg^−^ mode during host-to-host transmission, and maintain production of virulence factors required for reinfection. Thus, we speculate that similar to *B. bronchiseptica*, the Bvg^−^ mode serves *B. pertussis* cells to adapt to and survive in a hostile and nutrient-poor niche such as the intramacrophage environment. This concept is also congruent with other observations showing that Bvg^−^ mutants accumulate among persistent *B. pertussis* cells within the upper respiratory tract of experimentally infected rhesus monkeys [83] and that a spontaneous mutant of *B. pertussis* producing highly reduced levels of virulence factors was not attenuated in survival in primary human macrophages [84]. Moreover, several Bvg-activated genes were downregulated and, *vice versa*, several Bvg-repressed genes were induced during *B. pertussis* infection of the respiratory tract of mice [30,31].

Nevertheless, not all effects observed in intracellular cells can be simply explained by the shift to Bvg^−^ phase. First, we identified a large number of genes that were strongly modulated in internalized cells but were not reported as Bvg-dependent. Among these, we found genes encoding electron transport chain complexes, transport systems, ribosomal proteins, and various transcriptional regulators. Second, for some previously described Bvg-dependent genes, we observed either negligible change in expression or even opposite changes than expected. For example, the *btrS* gene encodes a transcription factor responsible for expression of the T3SS locus, which is highly upregulated in Bvg^+^ cells [9]. However, the expression of *btrS* was not affected at all in internalized cells, whereas the expression of almost the entire T3SS locus was strongly downregulated, as expected for Bvg-activated genes. Most strikingly, the operon required for lipid A remodelling (*BP0396–0399*) consists of Bvg-activated genes [9] and therefore, should be downregulated in the Bvg^−^ mode. Nevertheless, the expression of this locus was greatly increased in intracellular *B. pertussis* cells. Altogether, these observations indicate that other regulators besides the BvgAS system are involved in adaptation to the intramacrophage environment.

Overall, the data presented here demonstrate that *B. pertussis* cells actively adapt to the intramacrophage environment; suggest that the intracellularity of *B. pertussis* is in the context of its lifestyle; and expose the avirulent phase as an authentic phenotype of internalized *B. pertussis* cells.

## Supplementary Material

Supplemental Material

## Data Availability

RNA-seq data from the sequencing runs were deposited in the European Nucleotide Archive under project accession number PRJEB33395 and will be available immediately from the date of publication.
