# Supplementary material for: Avirulent phenotype promotes Bordetella pertussis adaptation to the intramacrophage environment
Source: Emerg Microbes Infect. 2023 Jan 19;12(1):e2146536. doi: 10.1080/22221751.2022.2146536 (PMC9858536; doi:10.1080/22221751.2022.2146536)
Supplement: Supplemental Material [file TEMI_A_2146536_SM4979.zip › Supplementary Table 2.docx]

**Supplementary Table 2. Strains and plasmid used in the study**

| **Strain or plasmid** | **Description** | **Reference** |
| --- | --- | --- |
| **Strains** |  |  |
| *Escherichia coli* |  |  |
| SM10(λ*pir*) | Maintenance and mobilization of pSS4245 vector | [1] |
| XL1 blue | Cloning strain |  |
| *Bordetella pertussis* |  |  |
| Tohama I | Wild-type strain | [2] |
| Δ*BP2871* | Mutant carrying markerless deletion of the *BP2871* gene | This study |
| Δ*BP3011* | Mutant carrying markerless deletion of the *BP3011* gene | This study |
| Δ*BP2871*Δ*BP3011* | Mutant carrying markerless deletions of *BP2871* and BP3011 genes | This study |
| **Plasmid** |  |  |
| pSS4245 | Conjugation vector for allelic exchange in *Bordetella pertussis* | [3] |
|  |  |  |

1. Simon, R., U. Priefer, and A. Pühler, *A Broad Host Range Mobilization System for In Vivo Genetic Engineering: Transposon Mutagenesis in Gram Negative Bacteria.* Biotechnology, 1983. **1** p. 784–791.

2. Kasuga, T., et al., *Studies on Haemophilis pertussis. III. Some properties of each phase of H. pertussis.* Kitasato Arch Exp Med, 1954. **27**(3): p. 37-47.

3. Inatsuka, C.S., et al., *Pertactin is required for Bordetella species to resist neutrophil-mediated clearance.* Infect Immun, 2010. **78**(7): p. 2901-9.
