# Supplementary material for: Avirulent phenotype promotes Bordetella pertussis adaptation to the intramacrophage environment
Source: Emerg Microbes Infect. 2023 Jan 19;12(1):e2146536. doi: 10.1080/22221751.2022.2146536 (PMC9858536; doi:10.1080/22221751.2022.2146536)
Supplement: Supplemental Material [file TEMI_A_2146536_SM4979.zip › Supplementary Table 3.docx]

**Supplementary Table 3. Primers used in this study**

| Purpose of the primers | Sequence (5′->3′)* |
| --- | --- |
| Construction of *BP2871* mutant (upstream region) | F: *CATACTAGT*CGGCCCCATCGTCTTCG  R: *GCCTTATAA***CAT**GTCTCCACCTTATTGATGCGA |
| Construction of *BP2871* mutant (downstream region) | F: *ATATTATAA***TGA**GGGCGGCCGCGGGCGCTA  R: *TCAGAATTC*GCTGGTGGAGCAGGAGACC |
| Construction of *BP3011* mutant (upstream region) | F: *TATACTAGT*TCCCACAGTTTCTCGAACGGC  R: *ATAGCTAGC***CAT**GGGATGGACGAATGCTGAC |
| Construction of *BP3011* mutant (downstream region) | F: *ATAGCTAGC***TAG**GCGCCGCCGTCAGTT  R: *ATAGAATTC*CGGCAACACCCTGCTGGTC |
| RT-qPCR analysis of *BP3501*(*vrg24*) expression | F: CTACGCGACCACGGCCAGCA  R: CGCCGCGCCCTTGTATGGAA |
| RT-qPCR analysis of *rbfA* expression | F: GCATGCCAAGGTGTATTTCACG  R: TGGGAACAGTATGGATGTGCAG |

*F, forward primer; R, reverse primer; sequences shown in italics indicate nucleotides added for cloning purposes including restriction enzyme recognition sites (underlined). The initiation and stop codons are shown in bold.
