# Supplementary figures and images for: Avirulent phenotype promotes Bordetella pertussis adaptation to the intramacrophage environment
Source: Emerg Microbes Infect. 2023 Jan 19;12(1):e2146536. doi: 10.1080/22221751.2022.2146536 (PMC9858536; doi:10.1080/22221751.2022.2146536)

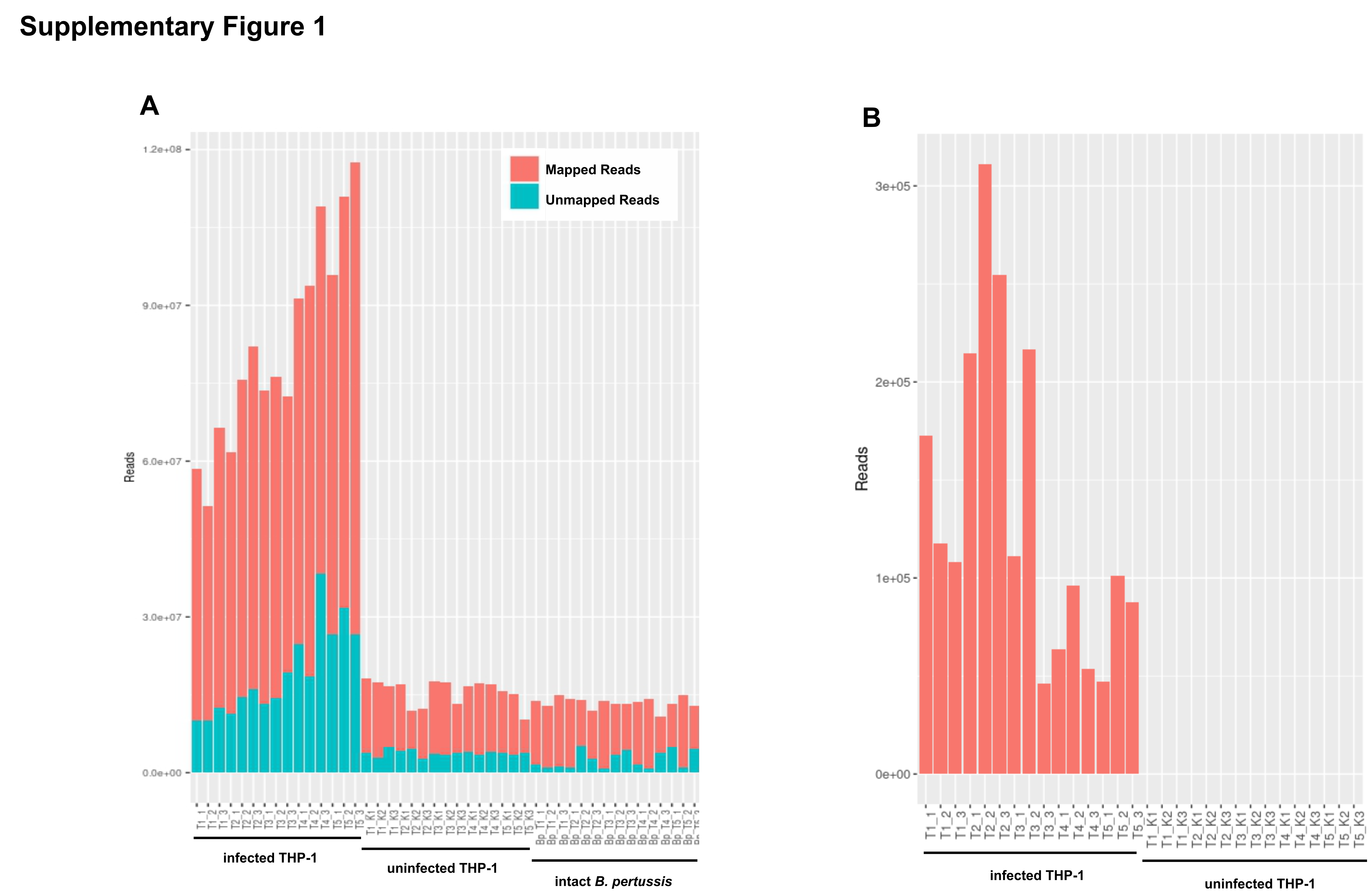

Supplement: Supplemental Material [file TEMI_A_2146536_SM4979.zip › Fig_S1.tif]

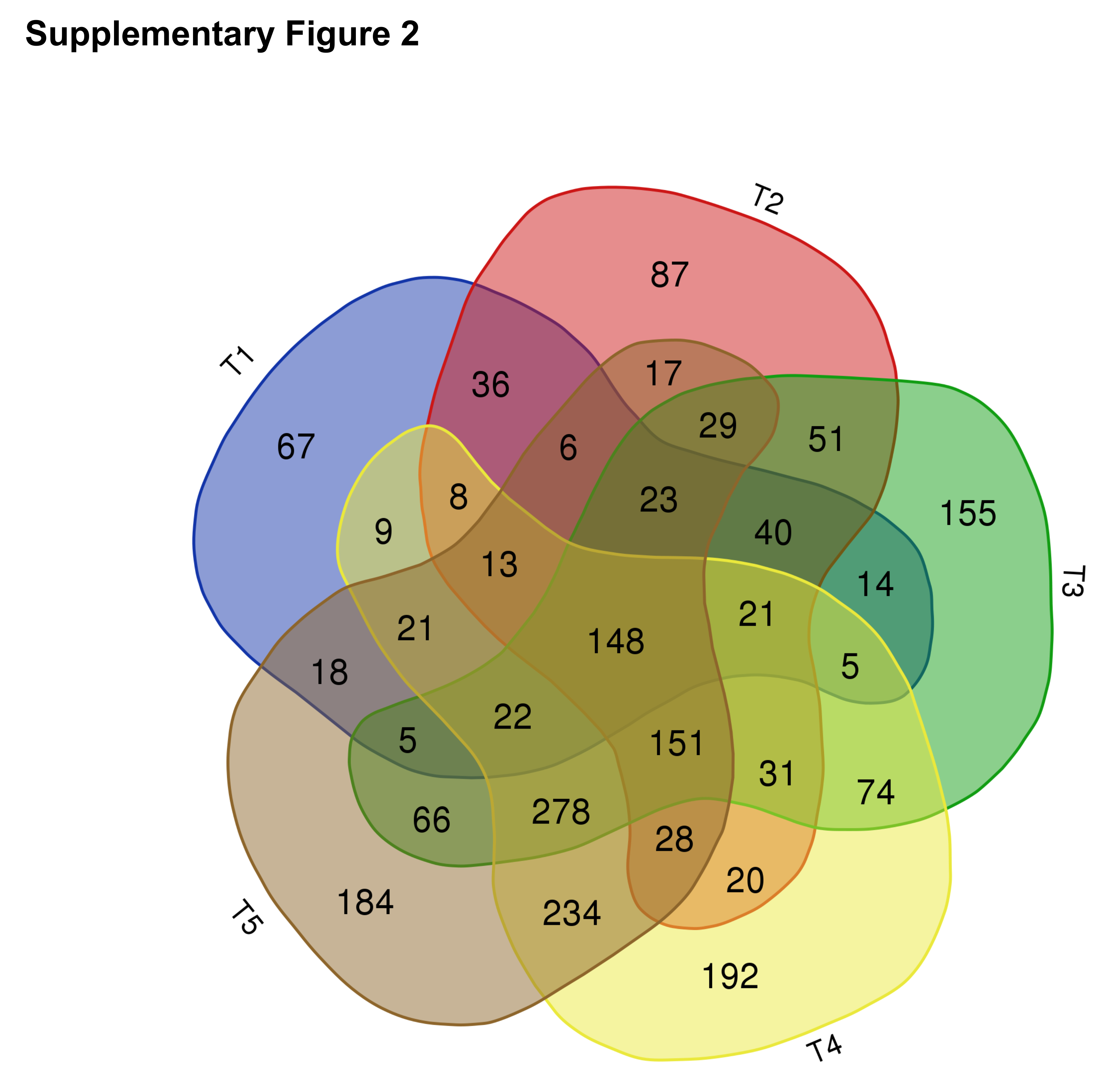

Supplement: Supplemental Material [file TEMI_A_2146536_SM4979.zip › Fig_S2.tif]

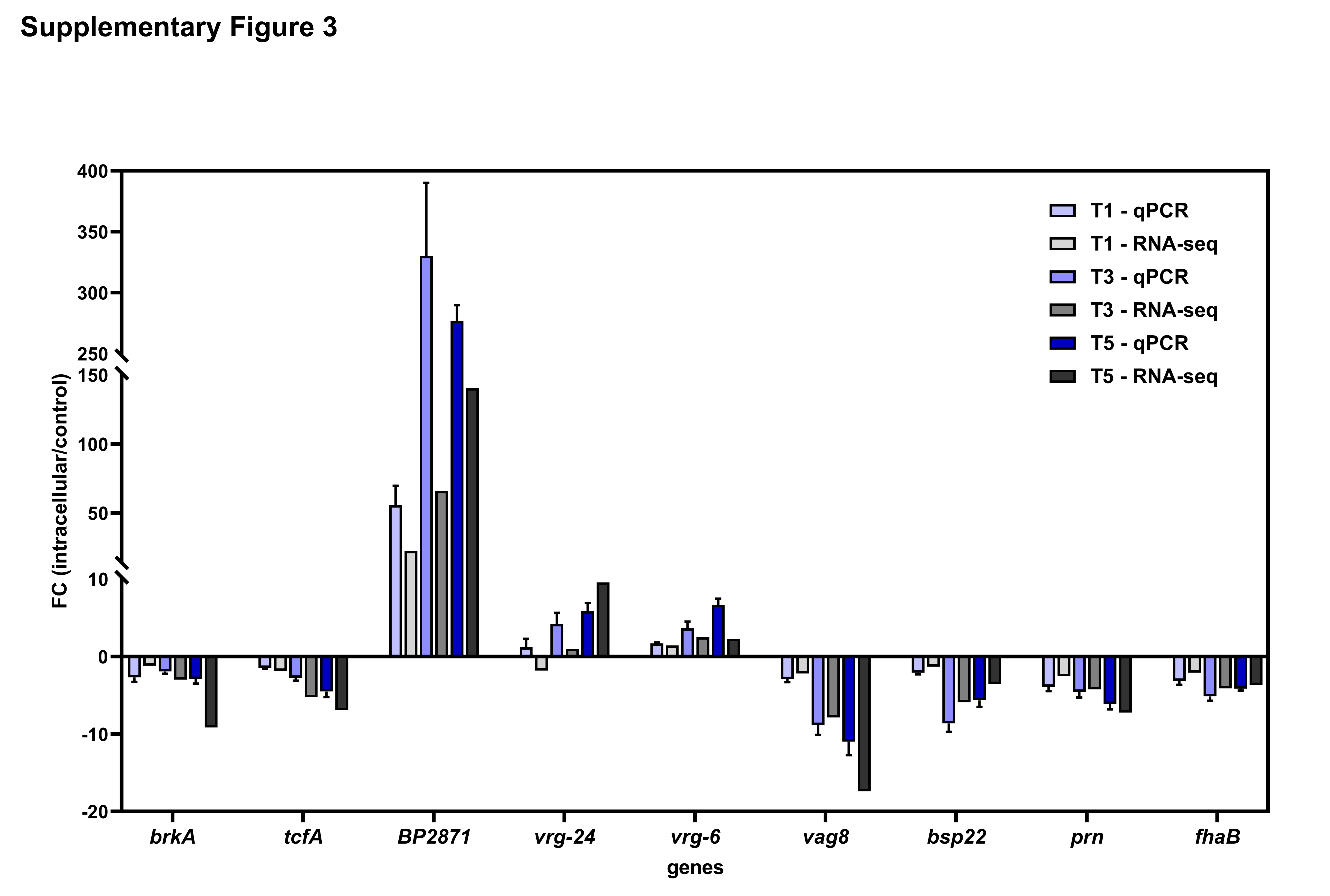

Supplement: Supplemental Material [file TEMI_A_2146536_SM4979.zip › Fig_S3.tif]
